# Infestation of shore crab gills by a free-living mussel species

**DOI:** 10.1007/s12526-016-0631-x

**Published:** 2017-01-24

**Authors:** Rowan Poulter, P. Graham Oliver, Chris Hauton, Trystan Sanders, Benjamin J. Ciotti

**Affiliations:** 1 Ocean and Earth Science, National Oceanography Centre Southampton, University of Southampton, European Way, Southampton, SO14 3ZH UK; 2 National Museum of Wales, Cardiff, CF10 3NP Wales; 3 Marine Ecology, GEOMAR Helmholz Centre for Ocean Research, Kiel, Germany; 4 School of Biological and Marine Sciences, Plymouth University, Plymouth, PL4 8AA UK

**Keywords:** Commensal, Infestation, Mussel-bound, Parasite, Predator–prey interaction, Shore crab

## Abstract

Parasitic and commensal species can impact the structure and function of ecological communities and are typically highly specialized to overcome host defences. Here, we report multiple instances of a normally free-living species, the blue mussel *Mytilus edulis* Linnaeus, 1758, inhabiting the branchial chamber of the shore crab *Carcinus maenas* (Linnaeus, 1758) collected from widely separated geographical locations. A total of 127 *C. maenas* were examined from four locations in the English Channel, one location in the Irish Sea and two locations at the entrance of the Baltic Sea. The branchial chambers of three crabs (one from the English Channel and two from Gullmar Fjord, Sweden) were infested with mussels resembling the genus *Mytilus*. Sequencing at the Me15/16 locus on the polyphenolic adhesive protein gene confirmed the identity as *M. edulis*. Bivalve infestation always occurred in larger red male individuals. Up to 16 mussels, ranging from 2 to 11 mm in shell length, were found in each individual, either wedged between gill lamellae or attached to the branchial chamber inner wall. This is one of the first reports of a bivalve inhabiting crustacean gills and is an intriguing case of a normally free-living prey species infesting its predator.

## Introduction

While parasitism and commensalism typically require a high level of specialisation, free-living non-specialists are occasionally found to colonise other species (Rohde 1984). For the coloniser this can offer both benefits, by increasing dispersal, providing access to food, removing waste products and offering protection (Key et al. 1996; Wahl 1989; Walker 1974), and disadvantages, by exposing them to stressful environmental conditions (Bruce 1989) and antifouling mechanisms of the host. Successful colonisation can also have negative implications for the host, such as reduced reproductive success and survival rates (Minchella 1985). Through impacts on host or coloniser populations, these instances of colonisation have the potential to influence the structure and function of ecological communities (Hatcher et al. 2006; Mouritsen and Poulin 2005; Poulin 1999; Poulin and Mouritsen 2006).

Certain taxa have managed to overcome anti-fouling grooming structures and behaviours in order to colonise the exoskeleton and branchial chambers of crustaceans (Bauer 1989). Commensal barnacles *Octolasmis* spp. infest branchial chambers and gills of numerous crustacean species (Gannon and Wheatly 1992; Santos and Bueno 2002; Walker 1974). Bopyrid parasites (e.g. *Pseudione* spp.) inhabit branchial chambers of decapod crustaceans (Boyko et al. 2012; McDermott 1991; Mori et al. 1999). Crustacean branchial chambers also host a variety of protozoa, helminths and crustaceans, most of which are small and highly specialised for survival and reproduction within the host (Shields 1992). However, aside from one observation of blue mussel *Mytilus edulis* Linnaeus, 1758, post-larvae on *Paralithodes camtschaticus* (Tilesius, 1815) gills (Jansen et al. 1998), we are not aware of any examples of bivalves inhabiting the internal structures or branchial chambers of crustaceans.

Bivalves, principally those belonging to the superfamily Galeommatoidea, certainly have the potential to associate with a range of invertebrates, including echinoids, holothurians, polychaetes, sipunculans, echiurids, brachiopods and crustaceans (Li et al. 2012). In some cases, bivalves inhabit internal structures of other organisms, such as the respiratory chamber of polychaetes (Rosewater 1984) or the oesophagus of holothurians (Bristow et al. 2010), or are embedded in the tissues of sessile organisms such as ascidians (Bodger and Allen 2008). Associations with crustaceans are mostly restricted to the burrows or undersides of burrowing forms such as *Upogebia* sp., *Squilla* sp. and *Lysiosquilla* sp. (Li et al. 2012). Isaeva et al. (2001) found that the free-living bivalves *Mytilus trossulus* Gould, 1850, and *Hiatella arctica* (Linnaeus, 1767) could be facultative epibionts on *Hemigrapsus sanguineus* (De Haan, 1835) when normal cleaning behaviour was interrupted by rhizocephalan parasites. Overall, however, association with decapod crabs is rare and, again, limited to external attachment (Boss 1965; Goto et al. 2007; Kato and Itani 1995; Kosuge and Itani 1994; Lützen and Takahashi 2003; Morton 1972). While bivalves are able to form commensal relationships with a wide range of hosts, this association is largely restricted to the Galeommatoidea, and there are few records of such interactions between Mytiloidea and other free-living invertebrates.

We provide one of the first documented examples of a bivalve inhabiting the branchial chamber of a brachyuran crustacean. We report multiple instances of the normally free-living *M. edulis* in the branchial chamber of the shore crab *Carcinus maenas* (Linnaeus, 1758) collected from widely separated geographical locations.

## Materials and methods

*C. maenas* were collected from seven sites between November 2014 and March 2015 (Table 1). Between 15 and 22 crabs were sampled from each location using baited lines or traps. Individuals from Menai Straits, Mudeford Quay, Swanwick Jetty, Weymouth Harbour and Newton’s Cove were stored in full-salinity aquarium tanks for a maximum of 2 weeks prior to examination. Individuals from Kiel Fjord and Gullmar Fjord were stored in ambient water from collection locations for a maximum of 5 weeks prior to examination.

**Table 1:** Summary of *Carcinus maenas* populations sampled and bivalve infestations observed

Site	Location	Date	Collection depth (m)	Collection method	No. crabs examined	Mean CW^a^ ± 1 SD (mm)	% Male	% Red^b^	No. crabs infested	No. bivalves
Swanwick Jetty, UK	50° 53' 16" N	Nov-14	0–1.0	Line	20	37.4 ± 7.8	55	5	0	0
	1° 17' 46" W									
Mudeford Quay, UK	50° 43' 30" N	Nov-14	0–1.5	Line	20	41.5 ± 9.3	40	40	0	0
	1° 44' 23" W									
Weymouth Harbour, UK	50° 36' 28" N	Nov-14	0–0.5	Line	15	37.8 ± 5.4	27	20	0	0
	2° 26' 59" W									
Newton’s Cove, UK	50° 36' 15" N	Nov-14	0–1.0	Line	15	49.0 ± 6.0	100	80	1	1
	2° 27' 01" W									
Gullmar Fjord, Sweden	58° 15' 28" N	Nov-14	0–15	Trap	20	69.0 ± 3.9	100	68	2	22
	11° 27' 28" E									
Menai Straits, UK	53° 13' 39" N	Dec-14	<4.0	Trap	15	57.6 ± 4.8	100	40	0	0
	4° 09' 18" W									
Kiel Fjord, Germany	54° 25' 22" N	Mar-15	0–2.0	Trap	22	57.7 ± 5.4	N/A	N/A	0	0
	10° 12' 09" E									

^a^CW = Carapace width

^b^Crab colouration classified as red or green

The mass, carapace width (CW), sex and any external signs of damage or disease were recorded for each crab. The colour of the legs, claws and carapace underside was also noted. The carapace was then carefully removed and gills were examined with the naked eye. The number and location of bivalves inhabiting the gill chamber were recorded. Samples of bivalves encountered were fixed in Bouin’s solution and stored in alcohol (Gullmar Fjord) or frozen at −80 °C (Newton’s Cove) for genotyping.

A small piece (*ca*. 0.1 mm^3^) of gill tissue from each attached mussel was extracted for genotyping using the Me15/16 locus on the polyphenolic adhesive protein gene, which is diagnostic for species of the genus *Mytilus* (Inoue et al. 1995). DNA was extracted using the DNeasy^®^ Blood & Tissue Kit (Qiagen, Manchester, UK) according to the manufacturer’s protocol. Formalin-fixed/alcohol-preserved tissue was washed twice in phosphate-buffered saline prior to DNA extraction. The target sequence was amplified by polymerase chain reaction (PCR) using sense primer Me15 5′- CCAGTATACAAACCTGTGAAGA -3′ and antisense primer Me16 5′- TGTTGTCTTAATAGGTTTGTAAGA-3′ (Inoue et al. 1995). PCR was performed with the GoTaq^®^ G2 Flexi DNA Polymerase kit (Promega, Southampton, UK). Reactions were set up in a 50-μl volume containing 1X GoTaq^®^ Flexi Buffer, 2.5 mM MgCl_2_, 0.2 mM deoxyribonucleotide phosphates mix, 0.5 μM of sense and antisense primers and 1.25 U of GoTaq^®^ G2 Flexi DNA polymerase. The reaction mix was pre-heated at 95 °C for 2 min, then subjected to 35 temperature cycles followed by a final extension step of 5 min at 72 °C. Each cycle consisted of 30 s at 95 °C, 30 s at 56 °C and 30 s at 72 °C. Twenty microlitres of each PCR product was subjected to electrophoresis (72 V for 40 min) against a 100-bp DNA ladder (New England BioLabs, Hitchin, UK) on 2% agarose/TAE gel containing 0.5 μg ml^−1^ ethidium bromide. Gels were visualised under UV radiation to reveal clear amplicons of ca. 180 bp. Amplicons were excised with a scalpel, extracted with a QIAquick^®^ Gel Extraction Kit (Qiagen) and eluted with 30 μl sterile water. Two microlitres of the extract was cloned using the pGEM^®^-T Easy Vector System (Promega) following the manufacturer’s protocol. Four successful transformants for each sample were grown overnight at 37 °C in Luria broth containing 100 μg ml^−1^ ampicillin. Cultures were centrifuged at 1000 *x G* for 10 min, and DNA was extracted from the pellet using a QIAprep^®^ Spin Miniprep Kit (Qiagen). Purified plasmids were subject to conventional Sanger sequencing using standard M13 primers (Source BioScience, Oxford, UK).

## Results

**Fig. 1:**
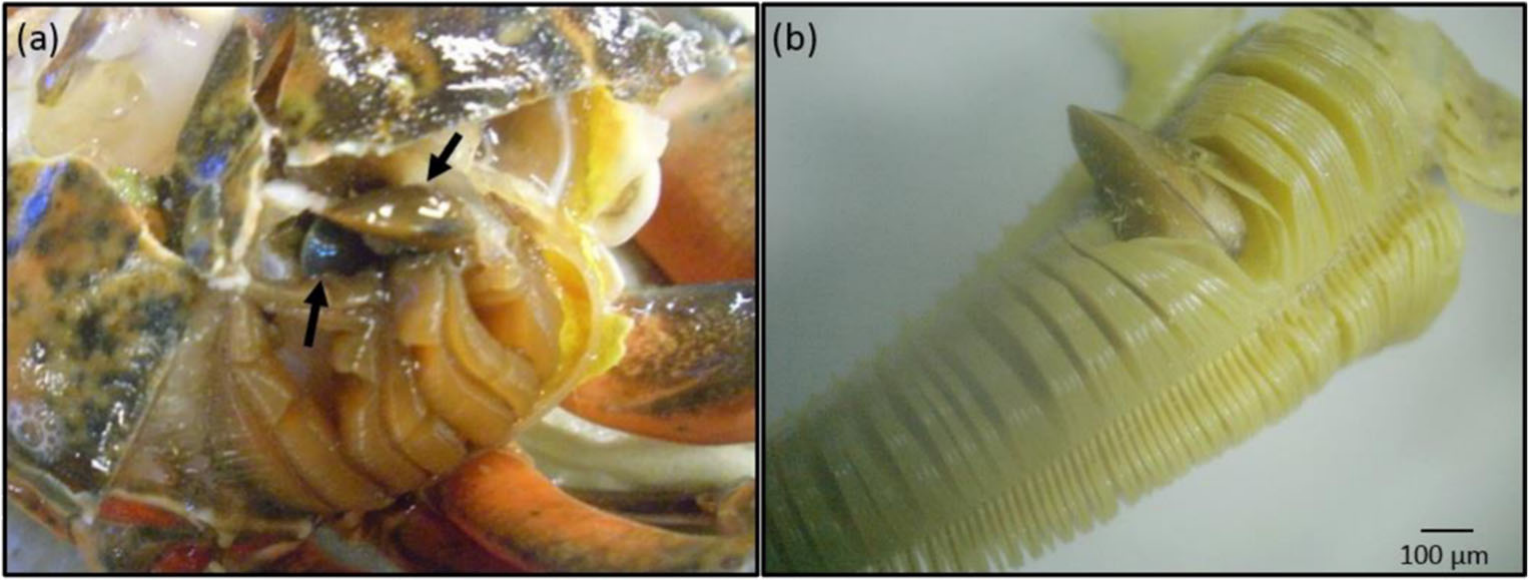
Photograph of *Mytilus edulis* infestations on *Carcinus maenas* gills from Gullmar Fjord: **a** view of *M. edulis* (*arrows*) in *C. maenas* branchial chamber after removing the carapace, and **b** detail of *M. edulis* embedded between gill lamellae

Bivalves were found in the branchial chambers of *C. maenas* from widely separated populations (Table 1). At Gullmar Fjord, Sweden, bivalves were found in two (out of 20 sampled) *C. maenas* individuals. One of the infested *C. maenas* contained six bivalves, while the second contained 16 bivalves and had died during holding. Bivalves were located in both right and left branchial chambers around the fifth gill, either wedged between gill lamellae or attached to the surrounding branchial chamber wall (Fig. 1). The size of infesting bivalves varied considerably within any one *C. maenas*, ranging from 2.0 to 11.0 mm in shell length. At Newton’s Cove, a single bivalve was found in one of the 20 *C. maenas* individuals sampled. This bivalve had a shell length of 6.7 mm and was wedged between lamellae on the posterior ninth gill. No bivalves were observed in the other five *C. maenas* populations examined (Table 1).

Many of the crabs inspected were missing limbs or bore signs of black spot disease, but mussel infestations did not appear to be particularly associated with disease or with other parasites, such as rhizocephalans. Infested crabs from both Newton’s Cove (CW = 60 mm) and Gullmar Fjord (CW = 74 mm and 80 mm) were the largest crabs captured at their respective sites. Infested crabs always had red colouration, although most crabs caught were this colour (Table 1). All infested crabs were male, as were all other crabs caught at the sites where infested crabs occurred (Table 1). Gills on which bivalves were attached appeared to be wasted and were entangled by byssal threads.

**Fig. 2:**
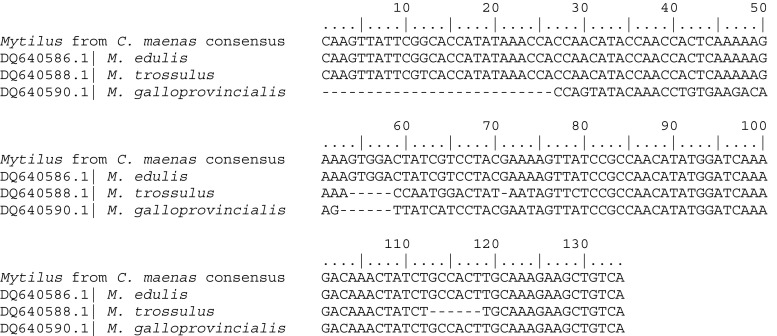
Clustal Omega sequence alignment (www.ebi.ac.uk/Tools/msa/clustalo) of a diagnostic region of the polyphenolic adhesive protein gene from bivalves found in *Carcinus maenas* against reference sequences for *Mytilus* congeners: *M. edulis*, *M. trossulus* and *M. galloprovincialis*. The consensus sequence represents 100% identity for four clones from two mussels found in the branchial chamber of *C. maenas* at Newton’s Cove and Gullmar Fjord. Reference sequences were originally published by Santaclara et al. (2006), and are listed with GenBank accession numbers.

Sequencing at the Me15/Me16 locus confirmed that infesting bivalves at both Gullmar Fjord and Newton’s Cove were *M. edulis*, rather than other locally occurring, morphologically similar congeners *Mytilus galloprovincialis* Lamarck, 1819 or *M. trossulus*. One mussel specimen from each of the two sites was genotyped. A 180-bp fragment was amplified in all cases, consistent with *M. edulis*, but not *M. galloprovincialis* (126 bp) or *M. trossulus* (168 bp) (Inoue et al. 1995). Consensus sequences for these four clones, generated through Clustal Omega alignment (www.ebi.ac.uk/Tools/msa/clustalo), were identical for the two mussel specimens, indicating genetic similarity between the mussels infesting Newton’s Cove and Gullmar Fjord crabs. A BLAST search (blast.ncbi.nlm.nih.gov/Blast.cgi) of this consensus sequence found a >99% identity (179 of 180 bases) to bases 1169 to 1348 of the *M. edulis* gene for polyphenolic adhesive protein (GenBank accession number X54422.1). Alignment of the sequence against diagnostic sequences for the three *Mytilus* congeners (Santaclara et al. 2006) indicated clear sequence homology with *M. edulis* over *M. galloprovincialis* or *M. trossulus* (Fig. 2).

## Discussion

We report multiple instances of *M. edulis* inhabiting the branchial chamber of *C. maenas. M. edulis* were found in three fully mature male *C. maenas* individuals, one from the English Channel and two from Gullmar Fjord. Colonising mussels varied in size, but included some adults. Mussels were found both on the inner carapace and attached directly to the gill, despite the suggestion by MacKenzie et al. (1974) that gill surfaces are an unsuitable site for mussel attachment. Our results, and similar observations of the pedunculate barnacle *Octolasmis mülleri* (Coker, 1902) on gills of the blue crab *Callinectes sapidus* Rathbun, 1896 (Walker 1974), suggest that crustacean gills are a viable attachment site for internal commensal species. To our knowledge, this is the first report of such an association between *M. edulis* and *C. maenas*.

Our observation adds to growing evidence that *M. edulis*, although typically a free living organism, sometimes colonizes other species. *M. edulis* larvae have been reported in the gill chambers of haddock *Melanogrammus aeglefinus* (Linnaeus, 1758) and cod *Gadus morhua* Linnaeus, 1758 (MacKenzie et al. 1974); however, upon close inspection, the mussels were found to be attached to a parasitic copepod, *Lernaeocera* sp., and not the gill surface itself (MacKenzie et al. 1974). Bruno (1987) later found post-veliger larvae, thought to be *M. edulis*, attached to and embedded in the gills of farmed Atlantic salmon *Salmo salar* Linnaeus, 1758. Post-larval *M. edulis* have been found in the red king crab *P. camtschaticus*, but details of the size of the mussels and the permanence of attachment to the gills were not documented (Jansen et al. 1998). Therefore, our observation establishes that gills of marine animals can be subject to substantial infestation by large mussels and is, to our knowledge, only the second documented example of a bivalve inhabiting crustacean gills.

Assuming that growth rates in branchial chambers are similar to those in the wild, the sizes of the mussels suggest they were from the previous spring spawning period occuring around March to May (Chipperfield 1953). *M. edulis* exhibits a two-stage settlement, with pediveliger larvae settling preferentially on filamentous substrata in high-flow areas, separate from any adults, before releasing and drifting to a suitable adult habitat (Bayne 1964; Eyster and Pechenik 1987). The veliger larvae most likely entered the branchial chamber accidentally in the inhalant respiratory current of *C. maenas* and temporarily adhered to the gills (Walker 1974). Only the larvae that immediately attached to the gills would remain in the branchial chamber, as reversal of the respiratory current, aimed to clean debris from the gills, would remove any unattached individuals (Walker 1974). Once the pediveligers metamorphosed (0.26–0.35 mm) and grew to the post-larval dispersal size (2.0–2.5 mm) (Bayne 1964), it is unlikely that they would be able to leave the crustacean branchial chamber. Alternatively, *C. maenas* could encounter drifting pediveligers in large numbers whilst feeding on mussel beds (Lane et al. 1985). The accidental inhalation of these post-larval forms could lead to entanglement of the byssus thread around the gills and subsequent new thread production and settlement, similar to the proposed settlement of *M. galloprovincialis* on the fish parasite *Mothocya epimerica* Costa, 1851, within the branchial chamber of *Atherina boyeri* Risso, 1810 (Öktener et al. 2014).

Settled pediveliger larvae in juvenile crabs are likely to be dislodged during ecdysis, before reaching maturity (Shields 1992), but the prolonged intermoult period of mature crabs would favour the development of infestations (Bauer 1989). New exoskeletons created during moults are always green in appearance, while red crabs will have spent an extended period in intermoult, increasing the chances of mussel colonisation (Styrishave et al. 2004). These red crabs are often the larger males (CW > 60 mm) (Reid et al. 1994), which reduce moulting frequency in order to devote energy to reproduction (Styrishave et al. 2004).

Sampling timing may explain why observations of *M. edulis* infestations in *C. maenas* are rare. *C. maenas* populations are commonly sampled during the spring, summer and autumn months while crabs inhabit shallow sublittoral regions (Atkinson and Parsons 1973). Much of this period would be prior to *M. edulis* settlement or when larvae are very small and easily overlooked. From late autumn, mature *C. maenas* populations migrate offshore (Atkinson and Parsons 1973), so any mature adults infested with *M. edulis* would not be sampled. Our sampling time of November to December allowed for the collection of mature adults just prior to offshore migration after spring-spawned *M. edulis* had sufficient time to grow.

While consequences of colonisation by *M. edulis* for *C. maenas* are unknown, it seems unlikely to provide advantages. Presumably, *M. edulis* does not feed directly on the tissue of the host, but the size and number of colonising mussels would create other problems. Attachment of *M. edulis* to the gill and surrounding chamber and waste produced by the mussels could reduce the respiratory efficiency by obstructing the ventilatory stream, impairing gill movements, reducing the exposed gill surface area and removing oxygen in the inhalant water (Walker 1974; Gannon and Wheatly 1992). *M. edulis* could also obstruct or functionally impair cleaning appendages, further reducing gill efficiency due to fouling (Santos and Bueno 2002) and increasing the likelihood of further infestation. Although moulting might occur when *M. edulis* are small, and could be an important defence against colonisation (Bauer 1989; Walker 1974), large infestations could present a considerable obstruction to moulting. The fact that the only deceased crab at Gullmar Fjord happened to be one of the two infested individuals suggests that consequences could be lethal for the host.

Mussels colonising *C. maenas* at Gullmar Fjord were highly aggregated in just two individuals. Unless growth rates of colonising mussels were highly variable, these aggregations did not originate from a mass colonisation event, since they consisted of a wide size range of individuals. Reproduction within the host seems unlikely, because this would require long adult residence times and successful retention of offspring. Instead, we suggest that aggregated distributions result from increased chances of subsequent settlement following previous colonisation. Initial colonisation could promote further settlement due to gregarious settlement behaviour (Petersen 1984) or interference with gill cleaning function. In this way, establishment of a single individual could result in further infestation and a rapid deterioration in fitness of the host.

Although *M. edulis* could be acting as a facultative commensal or parasite of *C. maenas*, it seems more likely that this is a case of accidental colonisation, with negative outcomes for the coloniser. Certainly, mussels would be well protected from predators inside *C. maenas* and can likely survive to maturity, which is normally attained during the first year of life (Seed 1969). Since *M. edulis* is a suspension feeder, food delivery could be enhanced by high flows associated with active irrigation of the gill surfaces by the crab. However, the extensive multi-stage life-cycle of *M. edulis* (Bayne 1964), coupled with the risks of moulting and mortality of *C. maenas* during large infestations, makes it unlikely that reproduction, and therefore fitness, would be improved over a free-living mode of life.

This is the first report of *M. edulis* colonising *C. maenas* branchial chambers and only the second time such an association has been documented between a bivalve and a crustacean. Our finding opens several future lines of enquiry. Because infestations are relatively rare, occurring in only 2.4% of individuals inspected, more extensive sampling of a greater number of crabs at each site would be necessary to accurately establish the abundance and prevalence of mussels in branchial chambers. Further work is also necessary to fully understand the physiological effects of the relationship, to determine the ability of both *M. edulis* and *C. maenas* to survive in the long term, and therefore to assess ecological consequences. Finally, the environmental conditions or biological scenarios that promote mussel infestations require investigation. The association between *M. edulis* and *C. maenas* could have important ecological implications and provides an intriguing example of a prey species infesting a predatory host.
